# GAPcare: the Geriatric Acute and Post-acute Fall Prevention Intervention—a pilot investigation of an emergency department-based fall prevention program for community-dwelling older adults

**DOI:** 10.1186/s40814-019-0491-9

**Published:** 2019-08-27

**Authors:** Elizabeth M. Goldberg, Linda Resnik, Sarah J. Marks, Roland C. Merchant

**Affiliations:** 1 Department of Emergency Medicine, Brown University, 55 Claverick Street, Providence, RI 02903 USA; 2 Department of Health Services, Practice and Policy, Brown University, 121 S. Main Street, Providence, RI 02912 USA; 3 Providence VA Medical Center, 830 Chalkstone Ave, Providence, RI 02908 USA; 4 Department of Emergency Medicine, Brigham and Women’s Hospital, Harvard University, 75 Francis Street, Boston, MA 02115 USA

**Keywords:** Care transitions, Emergency department, Older adults, Falls, Prevention, Medication therapy management, Physical therapy, Pharmacist, Pilot, Randomized controlled trial

## Abstract

**Background:**

Falls are the leading cause of fatal and non-fatal injuries among older adults. Older emergency department (ED) patients who present for evaluations after falls have a 30% higher risk of falling again in the subsequent 6 months than age-matched controls. Although EDs frequently evaluate older adults after their falls, the typical evaluation consists of an injury assessment alone. As such, an opportunity is lost to assess and address the potential causes of falls in this vulnerable population. In this manuscript, we present a multidisciplinary fall prevention protocol for a pilot study of older adult ED patients who recently sustained a fall (GAPcare: the Geriatric Acute and Post-acute Fall Prevention Intervention).

**Methods:**

GAPcare is a randomized single-blinded pilot study. Participants in GAPcare are 120 older adults (≥ 65 years old) who present to 1 of 2 academic US EDs after a fall. We randomly assign participants 1:1 to an intervention or a usual care (control) arm. In the intervention arm, the patient’s ED physician, a pharmacist, and a physical therapist (PT) collaborate to identify and address any risk factors that may have contributed to the fall. Intervention arm participants and their caregivers return home with a medication-related action plan to taper or stop potentially inappropriate medications and to address polypharmacy and a PT assessment and plan. Participants in the usual care arm receive standard assessments and care in the ED and a home safety brochure. Participants in both study arms complete fall calendars for 6 months to document the number of falls and healthcare visits during follow-up. The primary outcome is feasibility of the GAPcare fall prevention intervention (number and proportion of screened participants who are eligible, recruited, and retained; impact on ED length of stay), while the secondary outcome is to estimate its initial efficacy.

**Discussion:**

The GAPcare-ED fall prevention intervention has the potential to promote older adult-sensitive care for millions of Americans presenting to EDs after falls and establish a protocol for a future large-scale randomized controlled trial on this topic.

**Trial registration:**

ClinicalTrials.gov, NCT03360305. Trial registration date: December 4, 2017. Protocol version: 1

## Background

The Centers for Disease Control and Prevention estimate that by 2030, nearly one in five persons in the US will be ≥ 65 years old [1]. These older adults will comprise an increasing proportion of emergency department (ED) patients, and falls are the leading cause of fatal and non-fatal injuries that bring this population to the ED for evaluations [2]. ED evaluations for falls typically involve a focused assessment to rule out acute injuries, but do not identify modifiable risk factors to prevent subsequent falls [3–5]. ED patients rarely receive instructions to follow-up with clinicians that can address reasons for the fall [6]. Currently, only 3.7% of older adults receive fall guideline concordant care when they present to the ED after a fall [7]. According to the American Geriatrics Society and British Geriatrics Society guidelines, this care should include a multifactorial fall risk assessment that should be performed for all older persons who live at home and seek medical care in the ED because of a fall [8].

An interdisciplinary team of pharmacists and physical therapists (PTs) who perform standardized fall assessments is a key criterion for US geriatric ED accreditation [9]; however, currently, no applicable model exists to guide this care. Although falls are considered one of four high-yield research opportunities in geriatric emergency medicine [10], there is a notable lack of research in ED-based interventions to reduce the occurrence of recurrent falls among seniors. Preventing subsequent falls is critical to stop the cascade of functional decline, loss of independence, hospitalization, and death, which frequently follows a fall. Annual Medicare costs for adult falls are currently estimated to be $31.3 billion [11], and failure to find new, effective strategies to address falls in this population will lead to increased costs as the US population ages.

To address the lack of fall prevention research in the ED, we developed GAPcare (the Geriatric Acute and Post-acute Fall Prevention Intervention). This intervention brings together patients, caregivers, pharmacy and PT professionals, and clinicians to provide a patient-centric, collaborative approach to fall prevention that bridges the ED visit with outpatient resources. Performing a fall prevention intervention in the ED (as opposed to after the ED visit) provides more timely evaluation, which is of critical importance because seniors are at high risk of recurrent falls in the immediate post-fall period [12]. Unlike other current fall prevention protocols, this intervention starts immediately after the fall in the ED when patients and caregivers are highly engaged and motivated to prevent further fall occurrences [13]. The purpose of this manuscript is to describe the study protocol of the initial GAPcare investigation.

## Purpose and methods

The GAPcare study aims are to (1) examine the feasibility of the GAPcare intervention by assessing the number of participants who are eligible, recruited, and retained and measure the impact on ED length of stay (LOS); (2) determine if the GAPcare intervention (versus usual care) reduces subsequent falls and ED visits and hospital admissions in the 6-month follow-up; and (3) solicit feedback from participants on the utility and barriers of the GAPcare intervention and collect suggestions for improvement of the intervention. The rationale for completing this pilot trial prior to completing a definitive study powered for efficacy was to assess whether patients, caregivers, and clinicians were open to exploring prevention efforts with our pharmacists and PTs immediately after an injury, whether we could perform the intervention within the time constraints of a typical ED visit, and to obtain initial estimates of efficacy to help us determine the sample size for the subsequent definitive trial.

### Design of the GAPcare intervention and study protocol

This study is a two-site parallel group, single-blinded pilot randomized controlled trial (RCT). The hospital Institutional Review Board approved the study. We registered the trial at ClinicalTrials.gov (NCT03360305). The GAPcare intervention incorporates key elements of prior fall interventions [14–18]. The intervention was developed from these key elements and shaped by expert opinion from a team of geriatricians, health service researchers, geriatric-specialized PTs, and case managers, as well as an emergency medicine residency-trained pharmacist. This group of experts also comprise this pilot RCT’s research team and assisted in the creation of the study protocol.

### Setting

The study is being conducted at two main sites that belong to the same health system in Providence, Rhode Island: Rhode Island Hospital, an academic tertiary-care hospital, and The Miriam Hospital, an academic community hospital. The two participating study sites represent the ideal environment for this pilot study. Rhode Island Hospital is the only federally designated Level I Trauma Center in the state and has an annual ED census of 105,000. The Miriam Hospital provides care to a primarily community-dwelling geriatric population with a high injury and illness acuity and has an annual ED census of 73,000.

### Eligibility

Patients, their caregivers, and ED clinicians are recruited jointly to participate in the study. Study eligible patients are 65 years old or older, English-speaking, able to provide written informed consent (or have a legally authorized representative consent), and present to the ED after a fall. Participants must be community-dwelling or live in assisted or independent living communities. The treating clinician must intend to discharge the patient after their initial evaluation. Patients are excluded if their mental status is altered (i.e., intoxicated, agitated), they plan to leave the state in the ensuing 6 months (which limits retention for follow-up and engagement in treatment plans), are undomiciled, or cannot be reached by telephone.

### Recruitment and enrollment

Research staff review the ED Electronic Health Record (EHR) (Epic®) for ED patients presenting after a fall. Participants are recruited when PT and pharmacy specialists are available, Monday through Friday from 7 AM to 4 PM. The patient’s ED clinician is asked to confirm that he/she plans to discharge the patient home. If this outcome is likely, the research staff confirms eligibility with the patient, invites the patient to enroll, and asks them to provide written consent for participation. Caregivers and treating clinicians are also asked to consent to study participation.

We first assess each patient’s decisional capacity. For patients who score less than 4 on the Six-Item Screener [19] (a screen for cognitive impairment) but are interested in participating in the study, the legally authorized representative is asked to provide written consent.

After consent, each participant is randomized 1:1 to the intervention arm or usual care. Randomization is performed using REDcap and is based on randomization tables that stratify patients by study site. Enrollment staff do not have access to the allocation scheme before enrolling participants. It is not possible to blind the participants and treating clinicians to the allocation arm as they would be able to identify pharmacists and PTs at the bedside. However, the staff member who performs the assessment at 6-month follow-up is blinded to allocation arm.

### In-ED procedures

**Fig. 1:**
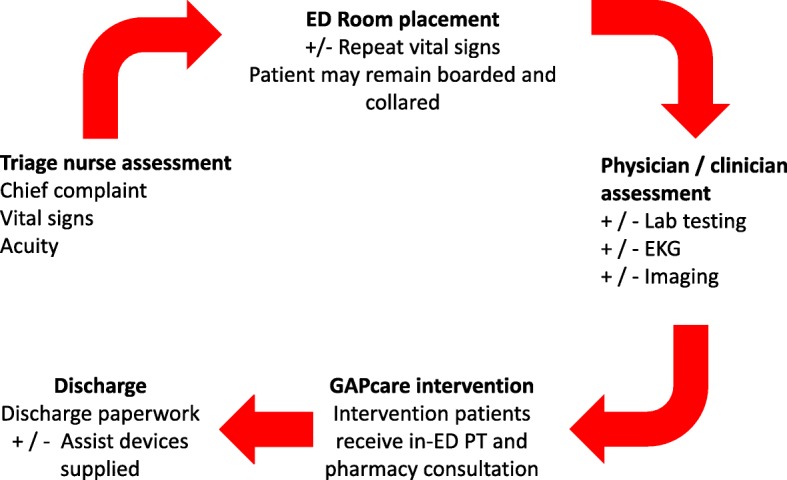
ED workflow and procedures for intervention participants. ED, emergency department; EKG, electrocardiogram; PT, physical therapy

After initial evaluation by the ED treatment team, patient participants are asked questions from the baseline questionnaire and their current medications are recorded by the research staff using previously published best practices for medication reconciliation [20]. Intervention patients receive individually tailored pharmacy and PT consultations according to the GAPcare protocol (see Table 1). Intervention participants receive all components of the intervention arm unless they refuse. The research staff delivers the intervention while participants are awaiting results of laboratory testing and imaging (see Fig. 1). Usual care participants receive care as dictated by the ED treatment team alone and a brochure from the Centers for Disease Control and Prevention about home safety. The research staff completes a timed up and go test on all participants regardless of study arm. All participants are provided with a fall calendar that allows them to record any new falls and healthcare visits at the time they occur for the following 6 months. It is at the discretion of the ED treatment team to contact the primary care physician (PCP), consult a case manager, and provide medical equipment (e.g., walkers, canes). Both intervention and usual care participants, their caregivers, and clinicians are asked to complete an end-of-visit survey to record their satisfaction with the care delivery, perceived barriers and facilitators, and suggestions for improvement.

**Table 1:** In-ED procedures for intervention patients

Personnel	Description	Administration
Pharmacist assessment and medication-related action plan		
Pharmacist	The pharmacist study protocol consists of the following steps: • Review the research staff obtained medication list [20]. • Perform motivational interviewing with the patient and/or caregiver to identify 1–3 medication recommendations, such as cessation or tapering of medication that increase fall risk. • Communicate the medication-related action plan (MRAP) in writing to the patient and ED treatment team. A facsimile copy of the MRAP is sent to the primary care provider (PCP) at the end of the visit via the newly created EHR structure.	• 20 min • In-person evaluation
Physical therapy assessment and action plan		
Physical therapist	The PT has the following responsibilities: • Performs a gait, balance, and lower extremity strength assessment. • Assesses the patient’s ability to function independently on discharge and assists with discharge planning. • Recommends outpatient services/referrals, such as referral to outpatient or home PT and occupational therapy, a home-safety evaluation, community fall prevention programs, or if necessary direct admission to a skilled nursing facility. • Communicates the PT action plan (PTAP) in writing and in person to the patient and ED treatment team. A facsimile copy of the PTAP is sent to the PCP at the conclusion of the visit via the newly created EHR structure.	• 20–30 min • In-person evaluation

*MRAP* medication-related action plan, *PCP* primary care provider, *PT* physical therapist, *PTAP* physical therapy action plan, *EHR* electronic health record

### Follow-up procedures

**Fig. 2:**
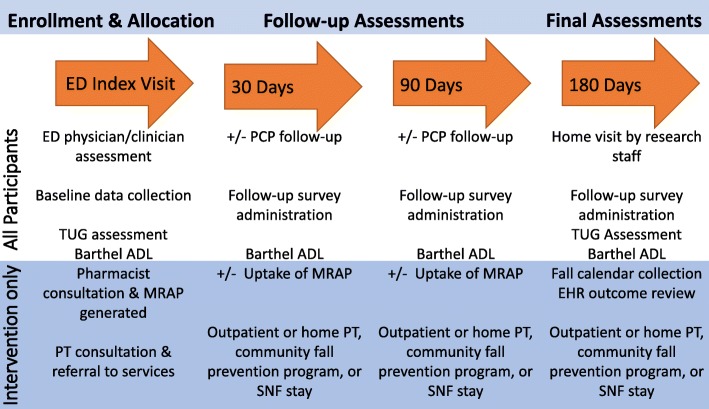
Schedule of enrollment, interventions, and assessments. PCP, primary care provider; TUG, Timed Up and Go test; Barthel ADL, Barthel Index for Activities of Daily Living; MRAP, medication-related action plan; SNF, skilled nursing facility

All participants receive telephone calls every 2 weeks to prompt them to complete fall calendars and provide them an opportunity to ask questions about study procedures (Fig. 2). At 30 and 90 days after enrollment, participants complete a follow-up survey over the telephone, via e-mail, or in person, depending on participant preference. At 180 days after enrollment, research staff, who are blinded to arm assignment, conduct a home visit with participants to perform the final procedures and assessments (Table 2). Fall calendars are collected at the conclusion of the visit. Research staff masked to arm allocation also abstract study outcomes from the EHR to confirm and supplement the data recorded in the fall calendars.

**Table 2:** Study instruments and timeline of assessments

Instrument	Description	Administration
Process evaluation		
Screening, eligibility, and retention	• Records how many patients were screened, agreed to participate, were recruited, received intended treatment, and were retained	• Baseline, 180 days
ED component and fidelity	• Records index visit ED LOS, time between consult call and arrival of pharmacist and PT, length of each consult, accuracy and completeness of action plan compared to the protocol	• Baseline
Patient, caregiver, and clinician feedback	• Tracks satisfaction with each component of the evaluation, perceived barriers and facilitators, and collects suggestions for improvement of the intervention	• Baseline
Follow-up component	• Tracks follow-up phone and in-person sessions with the research staff, PT, and any home services. Records uptake of recommendations made on the action plan for intervention participants	• 30, 90, 180 days
Outcome evaluation—feasibility, fidelity, and initial efficacy		
Enrollment questionnaire	• Demographic characteristics (includes marital status, whether the patient lives alone or with others, educational attainment, current employment.) • Prior fall history, comorbidities, ED index visit fall circumstances, and injuries	• Baseline • < 5 min
Six-Item Screener (SIS) [19]	• 6-point questionnaire to measure cognitive impairment for study screening [19], < 4 indicates high risk for cognitive impairment	• Baseline • 2 min
Timed Up and Go (TUG) test [21]	• Timed test of how long it takes the patient to get up from a chair, walk 10 feet, and return to the chair • Validated measure of current function, balance, and fall risk • A TUG > 12 seconds identifies those patients at greater risk for subsequent falls	• Baseline, 180 days • < 5 min
Barthel Index for Activities of Daily Living (ADL) [22]	• Used to assess functional independence [22] • Information can be gained by self-report or caregiver report	• Baseline, 30, 90, 180 days
Tinetti [23]	• Validated measure of gait and balance • Predicts falls	• Baseline • < 5 min
5 Times Sit to Stand [24]	• A measure of functional lower limb muscle strength	• Baseline
AM-PAC “6-clicks”	• Test of activity limitations and function [25] used to help determine need and intensity of outpatient services such as PT, safety of discharge, and to predict falls and healthcare utilization	• Baseline • 1 min
Falls Efficacy Scale-International [26]	• Measures fear of falling, which increases fall risk	• 180 days
Medication questionnaire	• Records all current and new prescriptions, over-the-counter and herbal medication, recommendations made by the pharmacist, recommendations accepted by the patient, and date of initiation/cessation	• Baseline, 180 days • 25 min
Outcome instrument	• Records follow-up falls and injuries by self-report and EHR review • Records subsequent ED visits and hospitalizations	• 30, 90, 180 days • 5 min

*ED* emergency department, *LOS* length of stay, *SIS* Six-Item Screener, *TUG* Timed Up and Go, *ADL* Activities of Daily Living, *PT* physical therapy, *AM-PAC* Activity Measure for Post-Acute Care, *EHR* electronic health record

### Efforts to improve recruitment and retention

A number of steps are taken to enhance recruitment and retention of participants. First, an Epic® Best Practice Advisory alerts research staff that a potentially eligible study subject is in the ED. Second, patients are asked during their index visit how they prefer to be contacted (by telephone or e-mail) and their preferred time of day for follow-up communication. Research staff schedule future follow-up telephone calls with patients during the 2-week reminder calls. If patients cannot be reached by telephone by the research staff, the principal investigator (PI) will attempt to contact them. Finally, an in-person visit is conducted for participants who cannot be contacted.

### Data collection

All participants receive an in-ED assessment by the research staff and scheduled follow-ups at 30, 90, and 180 days. Caregiver and clinician interviews occur only during the index ED visit. The information that is collected from participants and the EHR is summarized in Table 2.

We ensure fidelity to the research protocol through several means. First, the study coordinator reviews baseline enrollment data on a weekly basis. Second, the study team reviews on a monthly basis the proportion of subjects who are eligible, approached, consent to participate, and enrolled. Reasons for refusal are tracked to allow the study team to modify how they communicate study details when they approach the participant and for subsequent study planning. The PI holds weekly meetings with the research staff to discuss study progress and address concerns. Finally, the biostatistician performs automated and manual checks in the REDcap data collection program to ensure data quality.

### Sample size

This early stage investigation is designed to provide a preliminary indication of the proportion of screened participants who are eligible, recruited, and retained, with the goal of estimating these proportions with a standard error of less than 5%. Assuming the maximum possible variability for each of these proportions, the sample size of 120 produces a standard error of 4.6%, enabling estimation of a margin of error of ± 8.9%. A second feasibility aim was estimating if there was a major difference in ED LOS between groups. For *α* = 0.05 with a two-sided test and assuming a standard deviation of 120 min, a sample size of 52 per group has adequate power (*β* = 0.83) to detect a difference of 65 min. or more. To allow for non-parametric tests which may be necessary given the skewed nature of ED visits, a 15% increase in sample size or 60 per group will yield the same power [27].

### Data analysis

#### Feasibility

We collect the following feasibility measures and will report them descriptively [28]: number of patients screened, proportion eligible, number of patients recruited, time required to recruit, number of patients unable to provide consent, number of patient refusals, number of dropouts, and retention at each follow-up time point. We will use frequencies, proportions, rates, means/medians, and standard deviations and other measures of variability, as appropriate, to report on these feasibility measures. Also, we keep a record of lessons learned during the implementation of this study. To compare the median ED LOS between participants in both study arms, we will use the Wilcoxon-Mann-Whitney test and bootstrapping to generate a 95% confidence interval for the median difference in ED LOS. We will also examine the length of time required to complete the pharmacy and PT consultation in the intervention arm to determine if the intervention is feasible within the timeframe of an ED visit.

#### Fidelity

We will use descriptive statistics to assess key parameters of fidelity: receipt of the pharmacy and PT consultations, MRAP provision to patients and receipt of the MRAP by PCPs, fall calendar completion, and uptake of recommendations by patients and PCPs at 6-month follow-up. Implementation activities will be measured against recommendations and categorized into three level of adherence: adherent, partially adherent, and non-adherent.

#### Initial efficacy

We will complete the analyses on an intention-to-treat basis; participant outcomes will be analyzed according to their allocated arm, irrespective of the intervention received. Therefore, patients who are admitted or discharged to a skilled nursing facility after randomization will be included in the analysis.

We will compare the proportion and median/mean number of recurrent falls, ED visits, and hospital admissions over 6 months occurring in each arm using Fisher’s exact test (proportions) and Wilcoxon’s and Student’s *t* test (occurrence). If sample size permits, we will conduct a survival analysis for a time-to-event analysis. Survival time will be defined as the time from randomization to the time of the first recurrent fall. Dates and times for this outcome will be obtained from the fall calendar and EHR review. For this analysis, we will first use the Kaplan-Meier method to estimate time to the recurrent fall. Next, we will use the log-rank test to test differences between survival curves for the intervention vs. usual care arm. Finally, hazard ratios and 95% confidence intervals will be calculated using the Cox proportional hazards model; additional analyses will adjust for baseline differences in physical function. We may also use multivariate frailty models, which are an extension of traditional survival analyses that allow for multiple failures (in this case falls) and include random effects that take into account underlying individual predisposition to falling.

### Data and safety monitoring plan

Potential study-related adverse events or unintended effects will be reported to the data monitoring committee. This committee is composed of researchers who have no direct involvement in the study or direct relationship with the sponsor. The PI is ultimately responsible for ensuring participant safety throughout the trial period. An interim analysis is not planned. Protocol modifications will be reported to the Institutional Review Board.

## Discussion

GAPcare is a two-hospital, single-blind, randomized controlled trial that examines the feasibility of delivering an in-ED multicomponent fall prevention intervention with coordination of post-discharge services to community-dwelling older adults. This initiative is the first US trial to randomly assign patients who present with falls to a pharmacy and PT evaluation while they await routine care in the ED. This study is innovative for several reasons. Prior fall prevention studies have excluded patients with cognitive impairment and Parkinson’s disease, or those near the end of life. GAPcare includes patients regardless of dementia status, chronic disease burden, or prognosis. Therefore, our study results will be applicable to seniors who present to other academic medical centers. Although ED clinicians benefit from the team-based approach and expertise of the pharmacist and PT consults, ED clinicians are not called on to administer assessments or change their current ED workflow. This design allows for implementation of the study protocol without increasing clinician burden or requiring intensive pre-implementation education of ED physicians and staff.

The ED visit after a fall represents a “teachable moment” where patients are likely to recognize the need for prevention of future falls. Caregivers are often present and maximally engaged during this ED evaluation, as their loved one has just experienced an injury. The potential benefit of fall prevention is apparent to both the patient and the caregiver. Thus, we believe that this model addresses barriers to effective fall interventions identified in prior qualitative studies such as the denial of falling risk, the belief that fall prevention is not necessary, and practical impediments to attendance at follow-up appointments [29]. Because the intervention takes place in the ED, participants do not need to arrange transportation to complete the study. In addition, the immediate post-fall period is a high-risk time for older adults to have repeat falls [12]. This intervention is delivered prior to patients returning home, which is an ideal time to prevent falls in this high-risk period.

There are two main limitations of this study. First, this study is not designed or powered to measure a reduction in recurrent falls. A subsequent efficacy study will be performed with adequate power to test this aim. Second, both study sites are in a mid-sized urban area and the study results will not be generalizable to EDs that are in rural areas or EDs without availability of in-ED pharmacist or PT consultation. However, we anticipate that EDs with geriatric ED accreditation may use this model of care for their patients.

In the past decade, deaths from falls have increased by 30% and falls remain the leading cause of injury-related deaths in older Americans [30]. Falls are common, often preventable, and have a significant cost to the individual and society. The ED is ideally positioned to serve as a screening site for falls and to start prevention efforts. GAPcare pilots a new model of care that could prevent falls in the high-risk patient population that requires emergency care after a fall.

## Data Availability

Human subject protections preclude us from granting public access to participant-level data. However, aggregate results will be published in a peer-reviewed journal after study completion. Access to the full protocol and statistical code will be available after study completion.

## Abbreviations

AM-PAC: Activity Measure for Post-Acute Care
Barthel ADL: Barthel Index for Activities of Daily Living
ED: Emergency department
EHR: Electronic health record
EKG: Electrocardiogram
GAPcare: The Geriatric Acute and Post-acute Fall Prevention Intervention
LOS: Length of stay
MRAP: Medication-related action plan
PCP: Primary care provider
PI: Principal investigator
PT: Physical therapist
PTAP: Physical therapy action plan
SIS: Six-Item Screener
SNF: Skilled nursing facility
TUG: Timed Up and Go
